# Serum metabolites as predictive molecular markers of ovarian response to controlled stimulation: a pilot study

**DOI:** 10.5935/1518-0557.20190008

**Published:** 2019

**Authors:** Edson Borges Jr., Daniela Antunes Montani, Amanda Souza Setti, Bianca Ferrarini Zanetti, Rita de Cássia Sávio Figueira, Assumpto Iaconelli Jr., Diogo Oliveira-Silva, Daniela Paes de Almeida Ferreira Braga

**Affiliations:** 1 Fertility Medical Group. São Paulo, SP - Brazil; 2 Instituto Sapientiae - Centro de Estudos e Pesquisa em Reprodução Assistida. São Paulo, SP - Brazil; 3 Grupo de Bio-orgânica e Bioanalítica. Departamento de Química - Instituto de Ciências Ambientais, Químicas e Farmacêuticas – UNIFESP Diadema Campus, Diadema, SP - Brazil

**Keywords:** biomarker, controlled ovarian stimulation, ICSI, IVF, metabolomics

## Abstract

**Objective:**

This study aimed to look into the use of serum metabolites as potential biomarkers of response to controlled ovarian stimulation (COS) in patients undergoing intracytoplasmic sperm injection (ICSI) cycles.

**Methods:**

This case-control study analyzed serum samples from 30 patients aged <36 years undergoing COS for ICSI in a university-affiliated assisted reproduction center from January 2017 to August 2017. The samples were split into three groups based on response to COS as follows: poor responders: <4 retrieved oocytes (PR group, n=10); normal responders: ≥ 8 and ≤ 12 retrieved oocytes (NR group, n=10); and hyper-responders: >25 retrieved oocytes (HR, n=10). The metabolic profiles of the serum samples were compared between the groups through Principal Component Analysis (PCA). Receiver Operating Characteristic (ROC) curves were built to assess the power of the model at predicting response to COS.

**Results:**

PCA clearly distinguished between PR, NR and HR, and 10 ions were chosen as potential biomarkers of response to COS. These ions were more specific for PR than for NR. The ROC curve considering PR and NR had an area under the curve of 99.6% (95% CI: 88.9 - 100%).

**Conclusion:**

The preliminary evidence discussed in this study suggests that serum metabolites may be used as predictive molecular markers of ovarian response to controlled stimulation. The integration of clinical and “omics” findings may allow the migration toward an era of personalized treatment in reproductive medicine.

## INTRODUCTION

Controlled ovarian stimulation (COS) is a key step of ^in vitro^ fertilization used to stimulate the development of multiple follicles, enable the retrieval of multiple oocytes, and thus allow the selection of the best embryo for transfer. The retrieval of 5 -14 oocytes has been defined as a clinically appropriate ovarian response to COS (Kyrou *et al*., 2009), but poor and excessive response to COS are not rare.

The balance between retrieving too few or too many oocytes is difficult to strike, and may affect the chances of taking a baby back home and even increase the risk of patients suffering negative health outcomes through events such as the ovarian hyperstimulation syndrome (OHSS) (Kumbak *et al*., 2009).

Nevertheless, it is nearly impossible to accurately predict ovarian response and tailor stimulation protocols based on parameters such as maternal age, antral follicle count, anti-Müllerian hormone level, or prior poor/excessive response to COS. The development of noninvasive techniques to predict response to COS would allow individualized treatment, significantly increase treatment success rates, and alleviate the physical, emotional, and economic burden faced by patients.

Metabolomics is a great tool for the comprehensive study of the dynamic changes of the metabolome, and provides a powerful platform to discover biomarkers and improve diagnostic and therapeutic monitoring (Eckhart *et al*., 2012; Wang *et al.*, 2013; Hertel *et al*., 2016). In the field of assisted reproduction, most of the studies have focused on the embryonic metabolome through the analysis of spent culture media (Botros *et al*., 2008; Cortezzi *et al*., 2013). In addition, follicular fluid metabolites have also been studied to better understand oocyte competence (O'Gorman *et al.*, 2013), ovarian aging (de la Barca *et al.*, 2017), endometriosis (Santonastaso *et al.*, 2017), and polycystic ovarian syndrome (PCOS) (Zhang *et al.*, 2017). To date, there are no studies investigating the correlation between metabolomic profiling and response to COS.

This pilot study aimed to look into the use of serum metabolites as potential biomarkers of response to controlled ovarian stimulation (COS) in patients undergoing intracytoplasmic sperm injection (ICSI) cycles.

## MATERIAL AND METHODS

### Experimental Design, Inclusion and Exclusion criteria

This case-control study analyzed serum samples from 30 patients aged <36 years undergoing COS for ICSI in a university-affiliated assisted reproduction center from January 2017 to August 2017. The samples were split into three groups based on response to COS as follows: poor responders: <4 retrieved oocytes (PR group, n=10); normal responders: ≥8 and ≤12 retrieved oocytes (NR group, n=10); and hyper-responders: >25 retrieved oocytes (HR, n=10). The metabolic profiles of the serum samples were compared between the groups.

The patients gave informed consent prior to joining the study. The local institutional review board approved the study.

### Controlled Ovarian Stimulation and Laboratory Procedures

The patients were prescribed recombinant FSH (Gonal-F^®^, Merck KGaA, Darmstadt, Germany) for controlled ovarian stimulation and a GnRH antagonist (GnRH - Cetrotide^®^ Merck KGaA, Darmstadt, Germany) for pituitary suppression. Follicular growth was monitored using transvaginal ultrasound examination starting on day 4 of gonadotropin administration. When adequate follicular growth and serum E2 levels were observed, recombinant hCG (Ovidrel^®^, Merck KGaA, Darmstadt, Germany) was administered to trigger the final follicular maturation. The oocytes were collected 35 hours after hCG administration through transvaginal ultrasound ovum pick-up.

The retrieved oocytes were assessed to determine their nuclear status, and the ones in metaphase II were submitted to ICSI following routine procedures (Palermo *et al*., 1997).

### Sample Preparation, Metabolite Extraction, and Mass Spectrometry

Metabolites were extracted on ice for protein precipitation using Methanol/Chloroform (2:1 v/v). The mixture was centrifuged at 8,000×*g* for 15 min. After centrifugation, the supernatant was collected and transferred to a 96-well plate, which was placed inside a microTOF-QII^™^ mass spectrometer equipped with an Apollo II electrospray ion source (Bruker, Billerica, USA) coupled to a UFLC Prominence binary liquid chromatograph (Shimadzu, Kyoto, Japan).

The plate was stored into the SIL-30AC autosampler at 10ºC prior to analysis. The samples were directly injected into the analyzer in a 1 uL volume, by a 20 mmol/L ammonium formate solution in acetonitrile/2-propanol (4:1, v/v) at a flow rate of 200 µL/min.

Spectra were acquired in the positive mode using a range of *m/z* 50-1200 Da. Sodium formate clusters in isopropyl alcohol within the *m/z* 50-1200 Da range were used as the calibration standard.

### Data processing and statistical analysis

Patient and cycle characteristics were analyzed on SPSS Statistics 21 (IBM, New York, NY, USA). Variables were tested for normality and group homogeneity using the Shapiro-Wilk and Levenne tests, respectively. When needed, the samples were standardized using the z-score.

The variables were compared between groups through one-way ANOVA, followed by the Bonferroni post-hoc test. Variables were described as mean values ± standard deviation and significance was attributed when α was 5%.

Mass spectrometry results were obtained using the DataAnalysis 4.1 sofware (Bruker Daltonics Bremen, Germany) and data analyses were performed on MetaboAnalyst 3.0 (http://www.metaboanalyst.ca).

Log_2_ was used to normalize intensity values followed by self-scaling. Principal component analysis (PCA), an unsupervised method, was applied to the data set to detect intrinsic clusters based on metabolic profiles. From these analyses, a list of ions responsible for group discrimination was obtained.

These ions were used to build a receiver operating characteristic (ROC) curve and to evaluate the strength of the model at predicting response to COS.

Metabolite attribution was performed based on the Human metabolites database (http://www.hmdb.ca/), with a maximum mass tolerance of 0.01 Da. The maximum mass error was 30 ppm. For the attribution, only molecules containing hydrogen (M+H^+^), sodium (M+Na^+^) and potassium (M+K^+^) as adducts were considered.

## RESULTS

### Patient and cycle characteristics

The patient and cycle characteristics are described in Table 1. As expected, the level of estradiol on hCG trigger day, the number of aspirated follicles, the number of retrieved follicles, and the number of mature follicles were higher in the HR group followed by the NR group, while the PR group presented the lowest results.

**Table 1:** Patient and cycle characteristics for the poor, normal, and hyper-responder groups

	Poor responders (n=10)	Normal responders (n=10)	Hyper-responders (n=10)	*p*
Age (years)	33.88±1.87	32.40±2.75	31.30±2.11	0.065
BMI (kg/m^2^)	25.04±4.27	22.44±2.50	25.50±4.19	0.264
FSH dose (IU)	2383.33±668.48	2550.00±469.87	2495.00±584.38	0.825
Estradiol level (pg/ml)	913.00±415.80^a^	1818.00±1073.19^b^	3901.00±70.74^c^	*0.003*
Aspirated follicles (n)	3.50±0.85^a^	11.20±1.03^b^	50.40±22.24^c^	<*0.001*
Retrieved oocytes (n)	2.80±0.92^a^	9.70±1.49^b^	36.50±4.45^c^	<*0.001*
Oocyte retrieval rate (%)	82.50±23.71	86.82±11.84	81.03±24.98	0.816
Mature oocytes (n)	2.40±0.84^a^	7.30±1.56^b^	23.80±5.09^c^	<*0.001*

BMI= body mass index.

a ≠ b ≠ c (one way ANOVA followed by Bonferroni post hoc test, *p*<0.05)

### Metabolomic analysis

**Figure 1:**
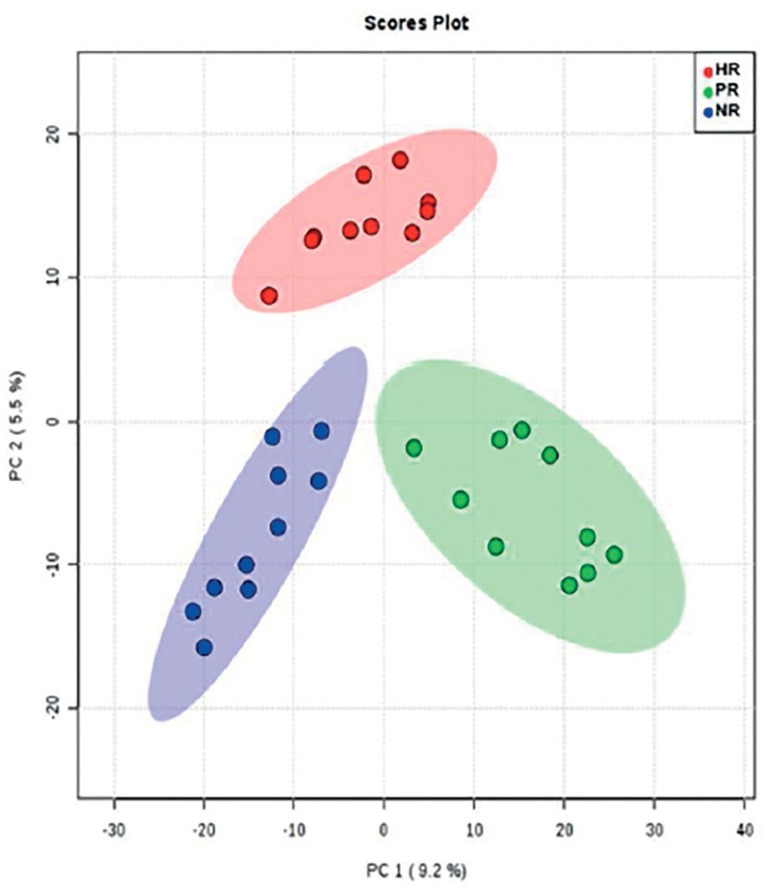
Variance among groups according to principal component analysis (PCA score plot)

Considering components 1 and 2, PCA clearly distinguished between the PR, NR, and HR groups (Figure 1).

The ion masses associated to the separation of the groups were obtained by the PCA loading plot, from which 10 ions were chosen as potential biomarkers (Table 2).

**Table 2:** Tentative biomarker attribution, loading values, and predictive values for high and poor responders

*m/z*	Loading	ROC (HR)	ROC (PR)	Adduct	Error (ppm)	Attribution	Formula
533.51975	0.06	0.61	0.75	M+Na^+^	13	Fatty alcohols	C34H70O2
685.43505	0.05	0.67	0.95	M+NH4^+^	1	Amino acids, peptides, and analogues	C29H57N5O12
880.7391	0.05	0.61	0.90	M+NH4^+^	24	Quinone and hydroquinone lipids	C59H90O4
631.3793	0.05	<0.5	0.80	M+Na^+^	4	Steroidal glycosides	C34H56O9
698.5985	0.05	<0.5	0.80	M+NH4^+^	16	Quinone and hydroquinone lipids	C48H72O2
522.5934	-0.06	0.54	0.81	M+H^+^	7	Tertiary amines	C36H75N
698.6051	-0.06	0.78	0.88	M+NH4^+^	26	Quinone and hydroquinone lipids	C48H72O2
876.79465	-0.05	0.53	0.73	M+NH4^+^	8	Triradylcglycerols	C55H102O6
880.7497	-0.05	0.57	0.81	M+NH4^+^	12	Triradylcglycerols	C56H94O6
296.2295	-0.05	0.72	0.94	M+NH4^+^	25	Methoxyphenols	C17H26O3

**Figure 2:**
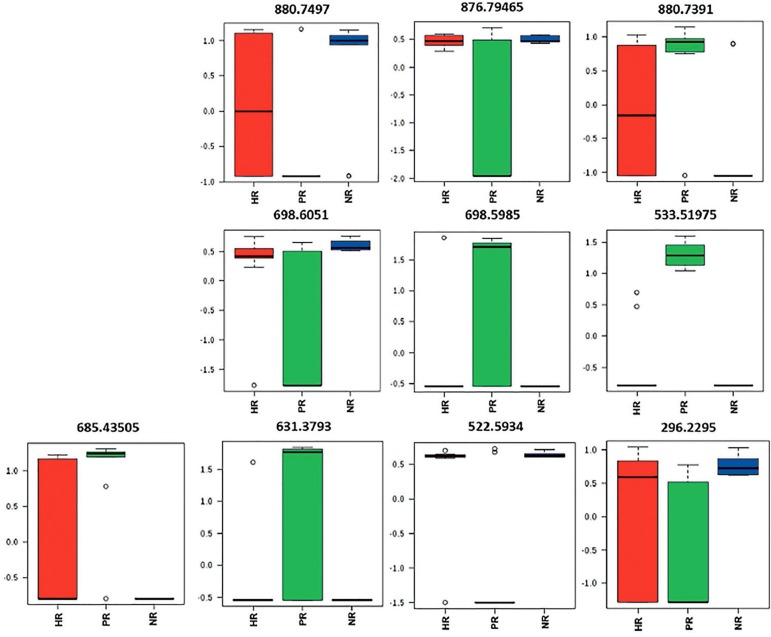
Boxes plot charts for the relative abundances of individual biomarkers

The relative abundances of individual biomarkers are demonstrated in the box plot charts (Figure 2). The PCA loading values for the selected ions based on principal component 1 are presented in Table 2.

**Figure 3:**
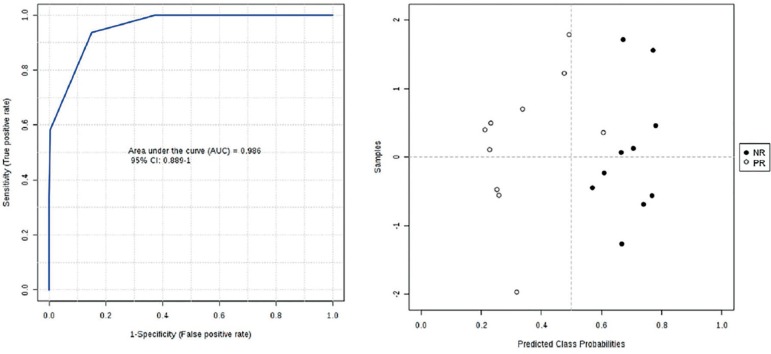
ROC curve and predicted class probabilities for normal and poor responders

Our evidence demonstrated that all ions selected in the present study were more specific for the PR group when compared to the NR group. The ROC curve considering the PR and NR groups presented an area under the curve (AUC) of 99.6% (95% CI: 88.9 - 100%, Figure 3).

## DISCUSSION

The success of assisted reproductive technology (ART) treatments is highly dependent on the response to COS. Moreover, considering the physical, emotional, and economic burden faced by patients undergoing ART treatment, finding a method to predict ovarian response to COS would be a major step forward in the field of reproductive medicine.

This study was able to identify metabolites that might be used as predictive molecular markers of response to COS. PCA clearly distinguished between PR, NR and HR groups, and 10 ions were chosen as potential biomarkers of response to COS. To date, this is the first study to investigate whether blood plasma metabolites might predict ovarian response to COS. The advantage of the method used in this study is having knowledge of the patients’ ability to respond to gonadotropins before the start of stimulation. Previous metabolomic studies in assisted reproduction investigated the metabolic profile on seminal plasma (Deepinder *et al.*, 2007) and fluid (Gupta *et al*., 2011), embryo spent culture media (Cortezzi *et al*., 2013), follicular (O'Gorman *et al*., 2013) and endometrial fluid (Braga *et al*., 2017).

OMICS technologies study cellular events and interactions from deoxyribonucleic acid (DNA) and genes to metabolites in a global way. Although metabolomics was recognized as a separate area of science much later than the other “omics” - such as genomics, transcriptomics, and proteomics - it provides a powerful platform for the discovery of novel biomarkers. The main advantages of metabolomics are its familiarity to the actual phenotype and the number of possible low molecular weight bio-compounds (Yoshida *et al*., 2012).

In conclusion, our preliminary evidence suggests that serum metabolites might work as predictive molecular markers of ovarian response to controlled stimulation. We believe that the quality of emerging data will eventually allow the individualization of COS, and further studies will validate new biomarkers of ovarian response. To date, the technology and software around metabolomics are still developing as the human metabolome is being mapped. The integration of clinical and “omics” findings will eventually allow the migration toward an era of personalized treatment in the field of reproductive medicine.
